# Effect of Probiotic Supplements on Oxidative Stress Biomarkers in First-Episode Bipolar Disorder Patients: A Randomized, Placebo-Controlled Trial

**DOI:** 10.3389/fphar.2022.829815

**Published:** 2022-04-26

**Authors:** Cuirong Zeng, Yan Qiu, Sujuan Li, Ziwei Teng, Hui Xiang, Jindong Chen, Xiangxin Wu, Ting Cao, Shuangyang Zhang, Qian Chen, Haishan Wu, HuaLin Cai

**Affiliations:** ^1^ Department of Pharmacy, The Second Xiangya Hospital of Central South University, Changsha, China; ^2^ The Institute of Clinical Pharmacy, Central South University, Changsha, China; ^3^ Department of Psychiatry, and National Clinical Research Center for Mental Disorders, The Second Xiangya Hospital of Central South University, Changsha, China

**Keywords:** probiotics, purinergic metabolism, oxidative stress, mania, bipolar disorder

## Abstract

**Background:** Currently no study has examined the effects of probiotic administration on the symptoms of anxiety, depression, and mania, as well as their correlations with the biomarkers of oxidative stress in patients with bipolar disorder (BPD). The aim of this study is to determine the effects of probiotic supplementation on plasma oxidative stress-related biomarkers and different domains of clinical symptom in patients suffering from BPD.

**Methods:** Eighty first-episode drug-naive patients with BPD were recruited. The subjects were randomized to receive psychotropic drugs supplementing with either probiotic or placebo and scheduled to evaluate with follow-ups for clinical symptom improvements and changes in the oxidative stress biomarkers. The Hamilton Depression Rating Scale, Hamilton Anxiety Rating Scale, and Young Mania Rating Scale were used to assess the clinical symptomatology. The panel of plasma oxidative stress biomarkers were determined by ultra-performance liquid chromatography–mass spectrometry (UPLC–MS/MS) at baseline and for 3 months of follow-up, i.e., at post-treatment month 1, 2, and 3.

**Results:** After 3 months of intervention, decreased levels of plasma lysophosphatidylcholines (LPCs) were found in both placebo and probiotic groups. However, six other oxidative stress biomarkers (i.e., creatine, inosine, hypoxanthine, choline, uric acid, allantoic acid) increased in BPD patients after the two types of therapies. In addition, a positive correlation between changes of LPC (18:0) and YMRS scale was found in BPD patients and this association only existed in the probiotic group. Additionally, the mania symptom greatly alleviated (pretreatment–posttreatment, odds ratio = 0.09, 95%CI = 0.01, 0.64, p= 0.016) in patients who received probiotic supplements as compared with the placebo group.

**Conclusion:** The changes in plasma biomarkers of oxidative stress in patients with BPD have a potential to be trait-like markers, and serve as prognostic indexes for bipolar patients. Daily intakes of probiotics have advantageous effects on BPD patients with certain clinical symptoms, especially manic symptoms. The treatment may be a promising adjunctive therapeutic strategy for BPD patients in manic episode.

## Introduction

Bipolar disorder (BPD) is a complex and chronic mental disorder, affecting approximately 1–2% of the general population at some point in their lifetime (Fagiolini et al., 2013). Bipolar and related disorders including manic episode, depression episode, and cyclothymic disorder are characterized by recurring depressive and hypomanic states (Carvalho et al., 2020). Recent evidence suggests that dysfunction of the purinergic system may play an important role in pathophysiology and treatment of BPD (Machado-Vieira et al., 2008; McQuillin et al., 2009). In addition, the activity of purinergic metabolic cycle was associated with increased oxidative stress in BPD (Albert et al., 2014). Meanwhile, purines affect the activity of other neurotransmitters, including GABAergic, dopaminergic, serotonergic and glutamatergic systems. It is worth noting that these factors are involved in the pathophysiology of BPD (Machado-Vieira et al., 2002). Purinergic system biomarkers include the purine nucleotides adenosine monophosphate (AMP), adenosine triphosphate (ATP), xanthines, uric acid, and related metabolites (Ali-Sisto et al., 2016). Adenosine is metabolized into inosine, hypoxanthine, and xanthine by cabolization with the enzymes adenosine deaminase and xanthine oxidase. In addition, oxidized phosphatidylcholine (PC) has been considered as a prominent biomarker of oxidative stress (Nakanishi et al., 2009). In support, lysophosphatidylcholines (LPCs) converted from PC during oxidation of low-density lipoprotein (LDL) increased in schizophrenia (Cai et al., 2012) and BPD (Schwarz et al., 2008) as compared to healthy controls, indicating elevated level of oxidative stress. Among the biomarkers, uric acid, a pivotal antioxidant in human body, is the key end product of nitrogen metabolism. Interestingly, a meta-analysis indicated that the plasma uric acid level in BPD patients was higher than that of healthy controls, especially in manic/mixed phases (Bartoli et al., 2016). The therapeutic effects on BPD symptoms may be associated with changes of plasma uric acid. After 8 weeks of treatment, non-remission subjects were reported to show higher level of serum uric acid concentration than remission subjects (Chen et al., 2019). Thus, it is possible that higher vulnerability to bipolar disorder is characterized by increased uric acid levels as a trait marker, which even increased more during mania (Albert et al., 2014). Increased uric acid levels implies accelerated purinergic transformation and reduced adenosinergic transmission (Burnstock, 2008). Activation of adenosine A1 receptors contributes to inhibition of neurotransmitter release and cellular excitability. The detailed mechanism remains to be illucidated.

Normally, gut microbiota modulates functions of the nervous system, immune and endocrine system (Tognini, 2017; Zeng et al., 2021). Disturbances in gut microbiota are also related to many mental diseases such as BPD, suggesting that the altered bacterial genus may cause oxidative stress and inflammation in patients (Coello et al., 2019). At the same time, specific species of the gut microbiota in turn regulate host purine metabolism, affecting disease status (Chiaro et al., 2017; Yamauchi et al., 2020). A vast number of studies have demonstrated that probiotics play an essential role in healthy population and clinical patients. Probiotics improves depressive symptoms, biomarkers of inflammation, and oxidative stress through their effects on neuronal circuits and the central nervous system mediated by the gut-brain axis (Foster and McVey Neufeld, 2013). A recent study has found the probiotics containing *Lactobacillus acidophilus*, *Lactobacillus casei*, and *Bifidobacterium bifidum* regulate depressive symptoms in BPD patients (Akkasheh et al., 2016). However, a recent systematic review conducted by Fusar-Poli and others (Fusar-Poli et al., 2019), has shown that the efficacy of adjunctive nutraceuticals in BPD is inconsistent, though they seem to be largely devoid of relevant side effects. On top of that, improved mood status and oxidative stress by decreasing inflammation and oxidative stress markers and increased nitric oxide bioavailability were observed after 3 months of supplementation with probiotics for Alzheimer’s patients (Ton et al., 2020). Although probiotic administration containing *Lactobacillus rhamnosus* strain GG and *Bifidobacterium* had no apparent therapeutic effects on the psychopathology, it may help prevent common somatic symptom associated with schizophrenia after 14 weeks (Dickerson et al., 2014). Other than psychiatric disorders, probiotic supplementation has therapeutic effects on mental health of petrochemical workers (Mohammadi et al., 2016). By comparison, there is now relatively robust evidence in animal studies to prove that probiotics can change behavior and improve depressive symptoms through regulating critical neurotransmitters, reducing overall inflammation, as well as its antioxidant and free radical scavenging abilities (Wallace and Milev, 2017). However, the benefits of probiotics on symptom relief and reduction of oxidative stress in patients with BPD has not been assessed to date.

This article aims to evaluate the therapeutic effects of probiotics on different symptoms of BPD patients in a randomized, placebo-controlled trial, and clarify underlying mechanisms of purine metabolism in BPD pathophysiology. We measured the plasma levels of nine metabolites linked to oxidative stress and purine metabolism [i.e., creatine, inosine, hypoxanthine, choline, uric acid, allantoic acid and LPCs (16:0, 18:1, and 18:0)] to investigate: 1) the changes of plasma levels of oxidative stress biomarkers in BPD patients, 2) the efficacy of probiotics on allieviating symptoms of BPD patients, and 3) the relationship between mitigation of BPD symptoms and changes of biomarkers after administrating probiotics.

## Materials and Methods

### Participants

This study was approved by the Medical Ethics Committee of the Second Xiangya Hospital, Central South University (2018-067). All participants gave signed and informed consent before all research procedures started. This study was registered in Chinese Clinical Trial Registry (ChiCTR1900021379).

Eighty patients suffering from BPD were recruited from March 2019 to November 2019 in the Second Xiangya Hospital of Central South University. The subjects were recruited from outpatients or inpatients who visited the hospital spontaneously. Based on the medical history and treatment provided by the patients and their families, as well as past medical records, it was confirmed that the patients had not received any drug treatment for BPD before enrollment. The inclusion criteria were as follows: 1) subjects aged 16–50 years; 2) diagnosed with BPD for a current manic or major depressive episode or mixed phase by two independent senior psychiatrists according to the DSM-5 (American Psychiatric Association, 2013); 3) no previous and current use of any psychotropic drugs was confirmed by medical records and patient self-reports. All participants were Han Chinese.

**FIGURE 1:**
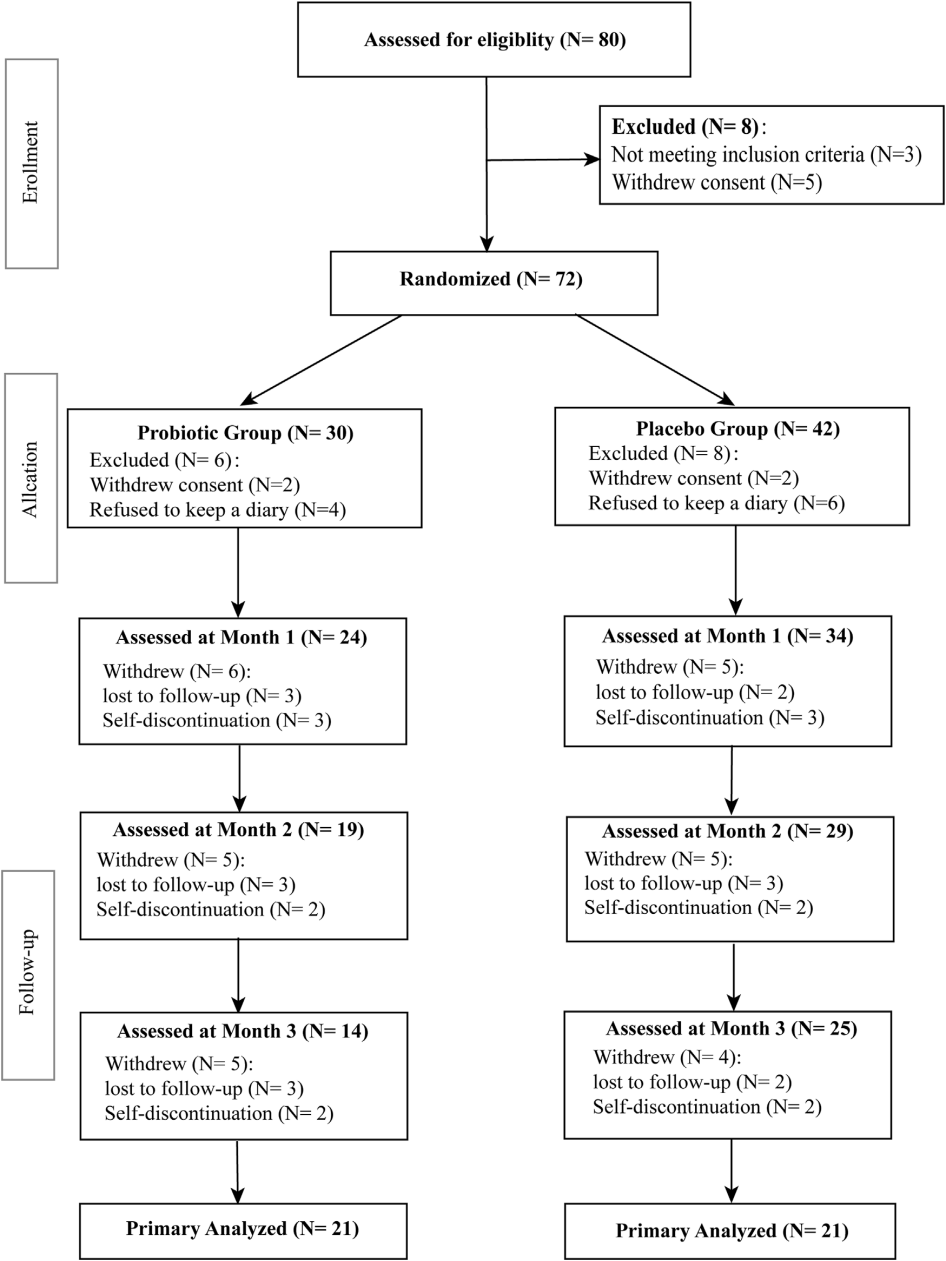
Flow chart indicating the recruitment of bipolar patients.

All subjects had no record of organic brain diseases, active or chronic inflammatory disease, cardiovascular disease, gout and renal disease. Patients’ exclusion criteria were comorbid mental disorders, intellectual disability, serious somatic diseases, autoimmune diseases and pregnancy or breast feeding. Subjects prescribed with drugs such as acetylsalicylic acid, thiazide diuretics, vitamin E, steroids, antiepileptic drugs or other drugs that may influence oxidative stress-related markers were also excluded. Seventy-two BPD patients finally met all inclusion criteria and none of the exclusion criteria were randomly assigned to the two intervention groups. The flow chart of the study design is shown in Figure 1.

### Intervention

This trial adopted second-level blind design. The first level of blind codes was the group corresponding to each subject number (such as group A and group B), and the second level of blind codes was the treatment plan for each group (such as test group and placebo group). The probiotic and placebo were produced by Shanghai Xinyi Pharmaceutical General Factory, and were numbered with groups A and B, respectively. Patients were assigned to receive either probiotic or placebo (without any restriction and stratification) by a computer-generated random number to ensure approximately equal numbers of patients within two groups. The treatment allocation was determined after all evaluations and study randomization. Patients were randomized to receive either probiotic (Live combined *Bifidobacterium*, *Lactobacillus* and *Enterococcus* capsules. Every capsule contains at least 1.0*10^7CF live probiotics for each capsule) plus psychotropic drugs or placebo plus psychotropic drugs for 3 months. Patients in the probiotic group were asked to receive three capsules after the meal twice a day at a fixed time, totaling six capsules daily. Patients in the placebo group were administrated with the placebo containing starch only. The appearance of the treatment products was indistinguishable in shape, package, size, color, taste, and smell to keep the treatment hidden to participants and researchers after allocation. Participants’ adherence to probiotic and psychotropic drugs treatment for each visit was defined as taking more than 80% and less than 120% of the pre-required medication dose specified for that interval. If participants did not comply, both patients and caregivers were informed the importance of taking prescribed doses of study drugs.

### Assessment

The team of clinicians and researchers was blinded to the information about the treatment group. Three rating scales were used for comprehensive assessment of symptom. The Hamilton Depression Rating Scale (HAMD) 17-item is a rating three- or five-point scale and is widely used to measure the severity of depressed symptoms (Hamilton, 1960). Hamilton Anxiety Rating Scale (HAMA) 14-item is a rating four-point scale used to measure the severity of anxiety symptoms (Hamilton, 1959). Young Manic Rating Scale (YMRS) is a 11-item multiple-choice diagnostic questionnaire used to measure the severity of manic symptoms (Young et al., 1978). Before conducting the study, five psychiatrists who had worked in clinical practice for more than 6 years simultaneously received training courses on how to use HAMD, HAMA and YMRS to ensure consistency and reliability of the scores. Repeated assessments of the study were performed to ensure that the correlation coefficient remained greater than 0.8. (Chen et al., 2019). All patients who received the treatment were scheduled for clinical assessment through each visit at baseline, months 1, 2 and 3. The baseline evaluation comprised demographics, anthropometric measures (body weight and height), physical examination, and comprehensive medical history. Severities of mood symptoms were measured in all subjects by HAMD, HAMA, and YMRS. During each follow-up, physical examination, anthropometry tests and three scales scores were repeated. A per-protocol analysis of the evaluable population was conducted to assess the clinical and metabolite outcomes. Only those patients who completed the study according to the intervention protocol specified at randomization and had complete metabolite measurements were included. The remaining patients were excluded from the analysis. The blood samples were collected from patients after fasting for 12 hours, using EDTA-coated vacuum tubes for plasma isolation. Plasma samples were obtained at baseline and post-treatment month 1, 2, and 3.

Plasma samples were immediately stored under −80°C until analysis. Concentrations of relevant biomarkers were measured using the ultraperformance liquid chromatography–mass spectrometry (UPLC–MS/MS) method of our research group (Cai et al., 2019).

### Statistical Analysis

The Kolmogrov–Smrinov test was used to test the normal distribution and homogeneity of variables. The independent *t*-test was employed to compare the variables at the baseline between the two groups. The two-way ANOVA (treatment and time as factors) analysis was conducted to compare the oxidative stress-related biomarkers concentrations, HAMD, HAMA and YMRS scores between the baseline and each time point in each patient group. The Pearson correlation analysis was performed to explore the relationship between oxidative stress-related markers levels and three scales scores. The ordinal logistic regression model analysis was carried out to assess the effects of probiotic supplementation on biomarkers of oxidative stress, YMRS scores, HAMD scores, and HAMA scores. The odds ratio indicated probiotics supplementation is a protective factor for the improvement of HAMD, HAMA, and YMRS. The treatment response was classified into two categories: poor response (Scale total score reduction from baseline<50%), and good response (Scale total score reduction from baseline≥50%) (Melander et al., 2003; Allgulander et al., 2004; Sachs et al., 2006; Hedayati et al., 2016). Results are expressed as mean ± SD. A two-tailed level of 0.05 was considered as statistically significant. All data were analyzed using SPSS (version 26.0).

## Results

### Demographic and Clinical Characteristics

Demographic and clinical characteristics of the subjects are shown in Table 1. No significant changes were observed in age (p = 0.5604), height (p = 0.2839), body weight (p = 0.9752), BMI (p = 0.9648), HAMD score (p = 0.5223), HAMA score (p = 0.4050), or YMRS score (p = 0.9670) within probiotic group and placebo group. As presented in Table 1, 17 patients were given mood stabilizers, 2 were given antidepressants and 14 were given antipsychotic medications in the probiotic group, whereas 21 were given mood stabilizers, 1 was given antidepressants and 18 were given antipsychotic medications in the placebo group. In probiotic group, 14 patients were in a depressive episode, 4 patients were in a manic episode, and three patients were in a mixed episode phase, while for the placebo control group, 15 patients were in a depressive episode, 4 patients were in a manic episode, and 2 patients were in a mixed episode phase. During the trial period, 38 patients withdrew from the trial and 42 patients completed the trial (Figure 1). Of 42 patients who completed the trial, 21 patients were randomly assigned to each group. There is no significant difference between the two groups (*p* > 0.05) in adverse events related to psychotropic drugs, including dizziness, somnolence, and nausea. These mild adverse reactions were alleviated or stabilized after treatment for 1–2 weeks. Meanwhile, no significant adverse effects occurred in subjects of the both groups.

**TABLE 1:** Baseline characteristics of the study participants.

Variable	Probiotic Group	Placebo Group	p
Patients	21	21	-
Age (years)	22.29 ± 5.13	20.86 ± 2.90	0.5604
Height (cm)	161.90 ± 5.57	164.81 ± 8.80	0.2839
Body weight (kg)	56.16 ± 7.38	58.38 ± 12.17	0.9752
BMI (kg/m^2^)	21.41 ± 2.61	21.37 ± 3.39	0.9648
Illness phase, n (%)			
Major depressive episode	14 (66.67)	15 (71.43)	-
Manic episode	4 (19.05)	4 (19.05)	-
Mixed phase	3 (14.29)	2 (9.52)	-
Psychiatric medication, n (%)			
Mood stabilizers	17 (80.95)	21 (100.00)	-
Antidepressants	2 (9.52)	1 (4.76)	-
Antipsychotic medications n (%)	14 (66.67)	18 (85.71)	-
Score of HAMD	20.10 ± 8.57	18.19 ± 10.04	0.5223
Score of HAMA	21.86 ± 8.16	19.38 ± 10.32	0.4050
Score of YMRS	10.29 ± 7.18	10.38 ± 7.28	0.9670

BMI, body mass index; HAMD, hamilton depression rating scale; HAMA, hamilton anxiety rating scale; YMRS, young manic rating scale; Data are mean ± SD; *p*-values were calculated using unpaired t-tests.

### Comparison of Oxidative Stress Biomarkers and Scales in Probiotic and Placebo Group After Treatment

**FIGURE 2:**
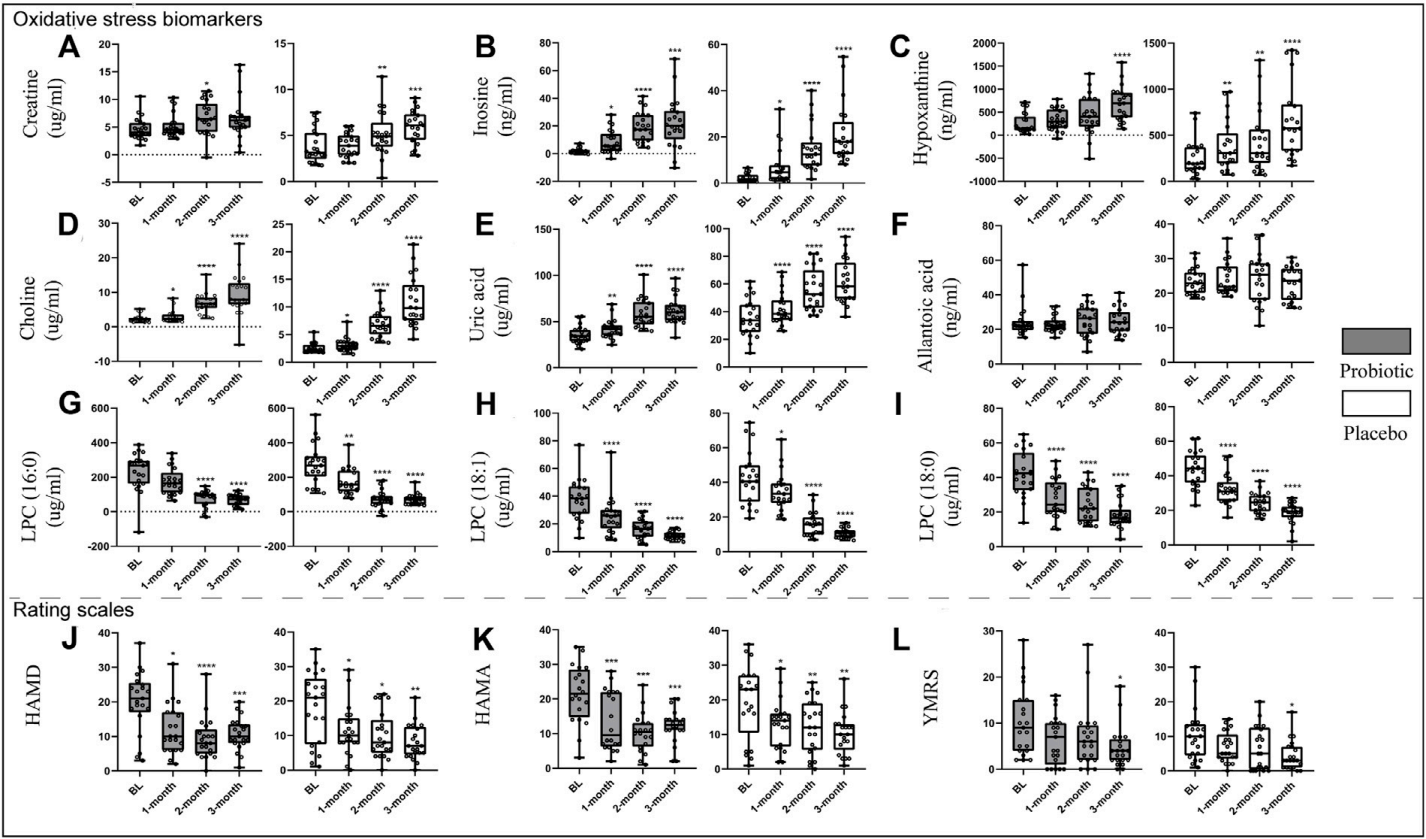
Changes in oxidative stress-related biomarkers [**(A)**, creatine; **(B)**, inosine; **(C)**, hypoxanthine; **(D)**, choline; **(E)**, uric acid; **(F)**, allantoic acid; **(G)**, LPC (16:0); **(H)**, LPC (18:1); **(I)**, LPC (18:0)] and scales [**(J)**, HAMD; **(K)**, HAMA; **(L)**, YMRS] after treatment in both groups. *p < 0.05, **p < 0.01, ***p < 0.001, ****p < 0.0001. Abbreviations: LPC, Lysophosphatidylcholines; HAMD, hamilton depression rating scale; HAMA, hamilton anxiety rating scale; YMRS, young manic rating scale.

Figure 2 displays an intuitive summary of the treatment effects over time. After 3 months of probiotic supplements concurrently used with antipsychotics medication, patients had significant increases in inosine (p = 0.0004), hypoxanthine (p< 0.0001) (Figures 2B,C), choline (*p* < 0.0001), and uric acid (p< 0.0001) (Figures 2D,E), while significant decreases in LPC (16:0) (p < 0.0001), LPC (18:1) (p< 0.0001), and LPC (18:0) (p < 0.0001) as compared to the baseline levels (Figures 2G–I), both in the probiotic and placebo groups. Additionally, patients had significantly lower HAMD (p = 0.0003), HAMA (p = 0.0001), and YMRS (p = 0.0150) scores as compared with their baselines (Figure 2J–L). However, there is no significant difference in improvements of oxidative stress biomarkers and symptomatology between probiotic and placebo groups.

In Table 2, it is indicated that probiotic supplementation is a protective factor for BPD patients. By comparing the probiotic groups with the placebo groups, the odds ratio was 0.62 for the rate of change in HAMD (pretreatment–posttreatment, 95% confidence interval [CI] 0.26, 1.47, p = 0.276), and 2.21 for the rate of change in HAMA (pretreatment–posttreatment, 95% confidence interval [CI] 0.71, 6.90, p = 0.173), without significance. The odds ratio for the rate change in YMRS between probiotic and placebo groups was 0.09 (pretreatment–posttreatment, 95% confidence interval [CI] 0.01, 0.64, p = 0.016), indicating that probiotic supplementation is a protective factor for the alleviation of manic symptoms.

**TABLE 2:** Outcome measures in a study of probiotic and placebo therapy in bipolar patients.

	Total sample (N = 42)		Probiotic Group (N = 21)		Placebo Group (N = 21)		Analysis		
Binary outcome measures	N	%	N	%	N	%	Odds ratio	95% CI	P
HAMD							0.62	0.26, 1.47	0.276
Rate of Change≥50%	26	61.90	13	61.90	12	57.14			
Rate of Change<50%	17	40.48	8	38.10	9	42.86			
HAMA									
Rate of Change≥50%	18	42.86	9	42.86	9	42.86	2.21	0.71, 6.90	0.173
Rate of Change<50%	24	57.14	12	57.14	12	57.14			
YMRS							0.09	0.01, 0.64	0.016
Rate of Change≥50%	26	61.90	14	66.66	12	57.14			
Rate of Change<50%	16	38.10	7	33.33	9	42.86			

HAMD, hamilton depression rating scale; HAMA, hamilton anxiety rating scale; YMRS, young manic rating scale.

Rate of change ≥50%, Good response; Rate of change <50%, Poor response.

### Correlations Between Oxidative Stress Biomarkers and Clinical Assessments

**FIGURE 3:**
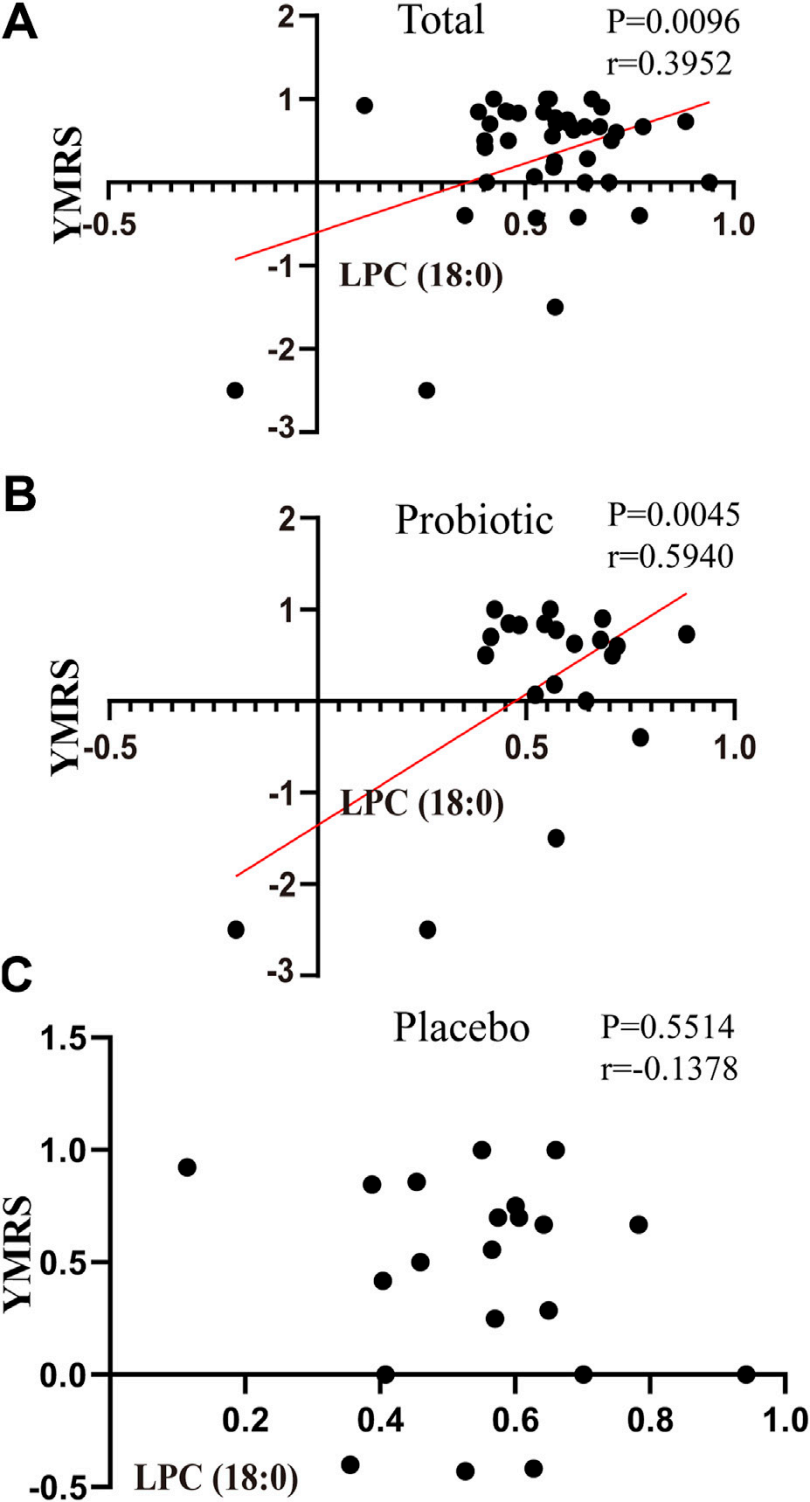
Correlations between the changes in oxidative stress-related markers and scales in placebo and probiotic groups after treatment. **(A)** the association between YMRS and LPC (18:0) in bipolar patients as a whole; **(B)** the association between YMRS and LPC (18:0) in the probiotic group; **(C)** the association between YMRS and LPC (18:0) in the placebo group. Abbreviations: LPC, Lysophosphatidylcholines; YMRS, young manic rating scale.

As shown in Figure 3, Pearson correlation analysis showed a significant correlation between LPC (18:0) concentrations and YMRS scores (pretreatment–posttreatment, r = 0.3952, p= 0.0096) in all BPD patients who completed the trail (Figure 3A). Specifically, a significant correlation between LPC (18:0) and YMRS index was seen in the probiotic group (pretreatment–posttreatment, r = 0.5940, p = 0.0045; Figure 3B), but not in the placebo group (pretreatment–posttreatment, r = -0.1378, p= 0.5514; Figure 3C).

## Discussion

The present study aims to assess oxidative stress biomarkers in BPD patients co-administrated with psychotropic drugs and probiotic/placebo. In the present study, decreased plasma LPC (16:0, 18:1, 18:0) levels in both placebo and probiotic groups were found. The other six oxidative stress biomarkers (i.e., creatine, inosine, hypoxanthine, choline, uric acid, allantoic acid) increased in BPD patients. Previous studies reported that the gut microbiota regulated purine metabolism (Chiaro et al., 2017). Of interest, purine metabolism relating to oxidative stress also plays a vital role in pathophysiology of BPD (Albert et al., 2014). Phospholipids belong to the most vulnerable class of biological targets of oxidative stress (Del Rio et al., 2005). The LPCs were converted from the oxidization of PC as markers of oxidative stress. Interestingly, the disturbance of lipid metabolism in patients with first-episode schizophrenia includes elevations of LPCs (16:0, 18:1, and 18:0), indicating an association with increase of oxidative stress (Cai et al., 2012). Uric acid, a purinergic metabolite produced by the xanthine oxidoreductase from xanthine or hypoxanthine, is an important nitrogenous end product of purinergic metabolism (ATP and adenosine) as well. Previous studies reported inconsistent data on blood levels of uric acid in BPD patients (Salvadore et al., 2010; Albert et al., 2014; Yang et al., 2018). Nevertheless, we observed increased uric acid levels in both treatment groups. Overall, changed plasma oxidative stress biomarkers, especially LPCs and uric acid, may serve as potential trait-like markers of prognostic values for BPD.

With regard to the role of gut microbiota in regulation of the immune system (Kamada et al., 2013), it is not surprising that imbalance of the intestinal ecosystem can increase inflammation and oxidative stress in the host (Nguyen et al., 2018), which may play a key role in the pathophysiology of BPD (Brown et al., 2014; Fernandes et al., 2016; Dickerson et al., 2018). The association of therapeutic outcome and the gut microbiota in patients with BPD was further explored, suggesting that the fractional representation of *Faecalibacterium* associated with better self-reported health outcomes (Evans et al., 2017). The association of increased fractional representation of *Faecalibacterium* with reduced depressive symptoms and improved physical health was also observed. Moreover, a recent study by Coello and others showed that *Flavonifractor*, a bacterial genus, may be associated with the pathogenesis of BPD by inducing host oxidative stress and inflammation (Coello et al., 2019). These results indicate that gut microbiota may be a new therapeutic target for BPD patients. Theoretically, supplementation of probiotics may mitigate oxidative stress and show a better outcome than placebo group. However, the add-on improvement did not occur in our study. Therefore, the possible influence of antipsychotics on the gut microbiota should be taken into account. Recently, the effects of the atypical antipsychotic drugs on the gut microbiome of 117 BPD patients have been examined in a cross-sectional design (Flowers et al., 2017). As compared with non-antipsychotic treated BPD patients, the BPD patients treated with antipsychotics were younger but had increased body mass index, and decreased species richness of the gut microbiota, suggesting that antipsychotic drugs have a negative impact in regulation of gut microbiota. Thus, the beneficial effects of probiotics may be partially neutralized by the co-administrated psychotropic drugs in our study.

The other purpose of the present study was to evaluate the effects of probiotic treatment on clinical symptoms of BPD patients. The results revealed that mania symptom in patients who received probiotic supplements alleviated more than that of the placebo group (Table 2). A significant reduction in mania symptoms observed in those BPD patients taking the probiotics reported by Dickerson and others was consistent with our findings (Dickerson et al., 2018). Their studies have shown that supplementation of probiotic prevents rehospitalization in patients with BPD, due to their anti-inflammatory effects through modulating the immune response (Dinan et al., 2013; Dickerson et al., 2018).

In addition, we also investigated the relationship between the improvement of BPD symptoms and changes of oxidative stress biomarkers after administrating probiotics. YMRS has primarily been used to evaluate the symptoms of mania in clinical trials. It is the main measure in the Systematic Treatment Enhancement Program for Bipolar Disorder study, which is the largest study to date on the effectiveness of treatment for BPD (Young et al., 1978). Notably, the improvement of YMRS scores presents a positive relationship with LPC (18:0) reductions in the probiotic group instead of the placebo group (Figure 3). LPCs are known to be a pathological component of oxidized LDL. It has been found that BPD patients experiencing a manic episode had significantly lower total cholesterol, high-density lipoprotein (HDL), and LDL than euthymic patients. The BPD patients have significantly lower total cholesterol and LDL levels in (hypo)manic than depressed patients (Fusar-Poli et al., 2020), indicating a greater oxidative stress in manic episode. As such, the decreased oxidation of LDL was related to the alleviation of manic symptoms in BPD. Our results emphasize the efficacy of probiotic supplements in specifically alleviating manic symptoms in BPD patients.

The underlying mechanisms of the therapeutic effects of probiotics on BPD symptoms are still unclear. Nonetheless, as mentioned earlier, there is evidence that probiotics may have anxiolytic effects itself (Foster and McVey Neufeld, 2013) by keeping the homeostasis of host microbiome (Wang et al., 2017). The absence of conventional gut microbiota influences the development of behavior accompanied by neurochemical alterations (such as N-methyl-d-aspartate, serotonin) in the brain as well (Neufeld et al., 2011). Another possible mechanism is that probiotics are able to reduce stress-induced corticosterone and the severity of BPD by improving the function of GABAergic system. However, similar neurochemical and behavioral effects were not observed in vagotomized mice. Interestingly, these results indicated that the vagus may be an important modulatory constitutive communication pathway between the gut microbiota and brain (Bravo et al., 2011).

There were limitations in the current study. First, the sample size was relatively small, especially only included BPD patients from one district of China. Further studies should include more patients from different districts to validate the findings. Second, the dietary profiles were not recorded, which could possibly influence precise analyses of the effects of probiotic. Third, the study of potential differences between the subtypes in plasma oxidative stress biomarkers was prevented, since bipolar I and bipolar II were not distinguished. Fourth, the drop-out rate is relatively high. Fifth, intention-to-treat (ITT) analyses was not performed. Finally, the modulatory effects of different types of psychotropic drugs on the composition of intestinal microbiota have not been investigated.

**FIGURE 4:**
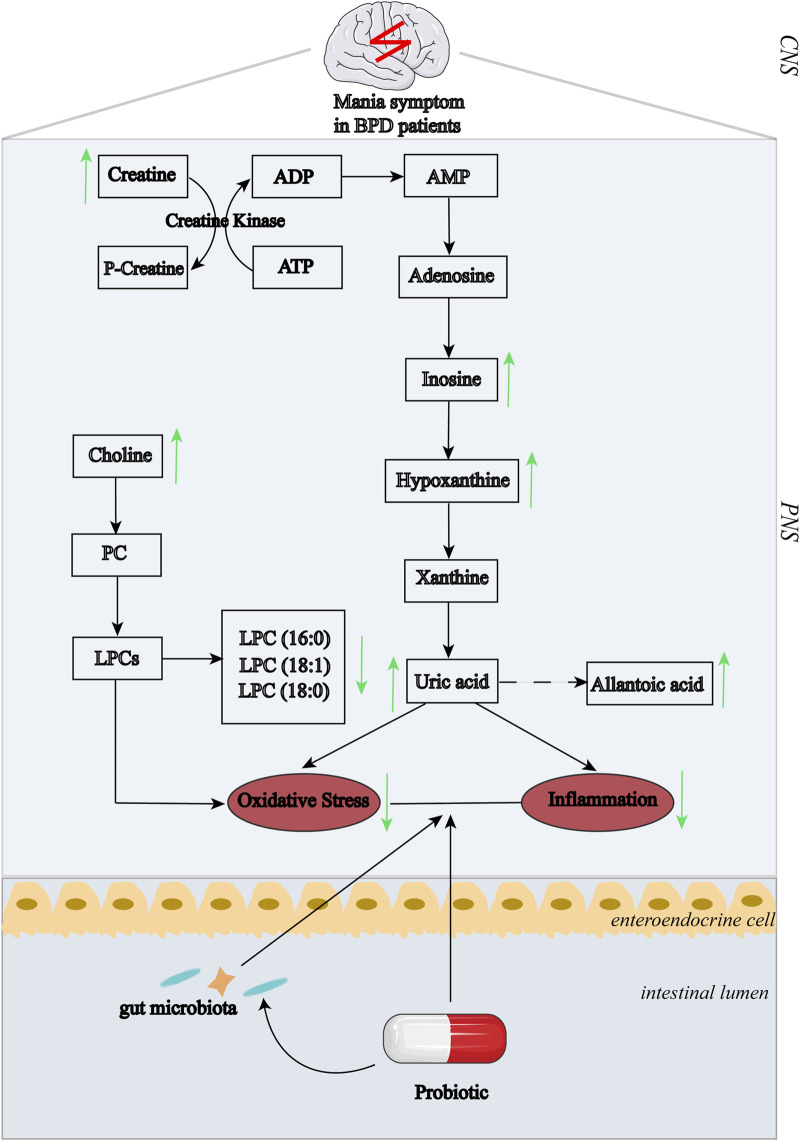
Summary of the associations between pathways of purinergic metabolism and BPD symptoms after probiotic treatment. The changing trend in response to treatment:↑, increase; ↓, decrease. Abbreviations: ADP, adenosine diphosphate; ATP, adenosine triphosphate; AMP, adenosine monophosphate; PC, phosphatidylcholine; LPCs, lysophosphatidylcholines; CNS, central nervous system; PNS, peripheral nervous system.

In conclusion, significant differences in oxidative stress-related biomarkers were determined in all BPD patients. Moreover, the probiotic supplementation specifically alleviated manic symptom as compared to the placebo group, possibly by regulating purine metabolism and reduction of oxidative stress status (Figure 4).

## Data Availability

The original contributions presented in the study are included in the article/Supplementary Material, further inquiries can be directed to the corresponding authors.
